# A Matter of Time: The Influence of Recording Context on EEG Spectral Power in Adolescents and Young Adults with ADHD

**DOI:** 10.1007/s10548-014-0395-1

**Published:** 2014-09-09

**Authors:** Glenn L. Kitsune, Celeste H. M. Cheung, Daniel Brandeis, Tobias Banaschewski, Philip Asherson, Gráinne McLoughlin, Jonna Kuntsi

**Affiliations:** 1 MRC Social, Genetic and Developmental Psychiatry Centre, Institute of Psychiatry, Psychology & Neuroscience, King’s College London, London, UK; 2 Department of Child and Adolescent Psychiatry and Psychotherapy, Central Institute of Mental Health, Medical Faculty Mannheim/Heidelberg University, Mannheim, Germany; 3 Department of Child and Adolescent Psychiatry, University of Zurich, Zurich, Switzerland; 4 Center for Integrative Human Physiology, University of Zurich, Zurich, Switzerland; 5 Neuroscience Center Zurich, University of Zurich, Zurich, Switzerland

**Keywords:** ADHD, EEG, Global field synchronization, Time, Context, Adolescents

## Abstract

Elevated theta or theta/beta ratio is often reported in attention deficit hyperactivity disorder (ADHD), but the consistency across studies and the relation to hypoarousal are increasingly questioned. Reports of elevated delta related to maturational lag and of attenuated beta activity are less well replicated. Some critical inconsistencies could relate to differences in recording context. We examined if resting-state EEG power or global field synchronization (GFS) differed between recordings made at the beginning and end of a 1.5 h testing session in 76 adolescents and young adults with ADHD, and 85 controls. In addition, we aimed to examine the effect of IQ on any potential group differences. Both regional and midline electrodes yielded group main effects for delta, trends in theta, but no differences in alpha or theta/beta ratio. An additional group difference in beta was detected when using regions. Group by time interactions in delta and theta became significant when controlling for IQ. The ADHD group had higher delta and theta power at time-1, but not at time-2, whereas beta power was elevated only at time-2. GFS did not differ between groups or condition. We show some ADHD-control differences on EEG spectral power varied with recording time within a single recording session, with both IQ and electrode selection having a small but significant influence on observed differences. Our findings demonstrate the effect of recording context on resting-state EEG, and highlight the importance of accounting for these variables to ensure consistency of results in future studies.

**Electronic supplementary material:**

The online version of this article (doi:10.1007/s10548-014-0395-1) contains supplementary material, which is available to authorized users.

## Introduction

Electrophysiological approaches provide a temporally precise method for recording electrical brain activity. They enable the direct investigation of subtle changes in cortical arousal, which are highly relevant for the study of attention-deficit/hyperactivity disorder (ADHD) where arousal dysregulation has been observed (Banaschewski and Brandeis 2007). Spectral electroencephalogram (EEG) is traditionally described as separate frequency bands: delta (0.5–3.5 Hz), theta (4–7.5 Hz), alpha (7.5–12.5 Hz), beta (12.5–30 Hz) and gamma (30+ Hz) (Tye et al. 2011). In control populations, compared to at-rest, cognitive tasks elicit reduction in alpha, suggesting that attenuation in alpha is associated with cognitive or attentional demands (Klimesch 2012). Similarly, increased arousal and attentional engagement through eye opening in the resting state not only induces global power reduction, but also topographic changes with decreases in frontal delta and theta activity, and frontal increases in beta activity (Barry et al. 2007), suggesting that the relationship between theta and beta activity may be an important marker of activation, while arousal seems more closely linked to global power and alpha activity reductions (Barry et al. 2007). Studies differ in whether data has been collected under eyes-open (EO) or eyes-closed (EC) conditions. Direct comparisons of EO and EC conditions in children and adults with ADHD suggest that EEG power differences are limited to an enhancement of alpha, and more tentatively, an attenuation of beta activity in the EC condition (Loo et al. 2009; Nazari et al. 2011; Woltering et al. 2012). Alternative group-level Independent Component Analysis (gICA) approaches, which may be more sensitive to spectral power differences than conventional techniques, identified reduced delta, alpha and beta voltage power and current source density in adults with ADHD compared to controls during both EC and EO conditions (Ponomarev et al. 2014).

The DSM-5 highlights that individuals with ADHD typically show increased slow-wave EEG (American Psychiatric Association. 2013). However, reported EEG spectral profiles in ADHD are far from consistent, and the extent to which these EEG indicators are useful in clinical settings remains unclear (Banaschewski and Brandeis 2007; Cortese 2012; Liechti et al. 2013).

The most consistent findings in earlier resting-state investigations of ADHD using both EO and EC data were of elevated theta or theta/beta ratio (T:B) in children, adolescents, and adults (Barry et al. 2010, 2009; Bresnahan et al. 1999; Clarke et al. 2001b, 2003b; Koehler et al. 2009; Lansbergen et al. 2011; Shi et al. 2012; Snyder et al. 2008; Woltering et al. 2012). Yet, some recent studies have failed to replicate these findings (Buyck and Wiersema 2014; Liechti et al. 2013; Loo et al. 2009; Ogrim et al. 2012; Poil et al. 2014; Ponomarev et al. 2014; Skirrow et al. paper under review; Swartwood et al. 2003; van Dongen-Boomsma et al. 2010), or have reported contrasting findings of attenuated T:B in adults with ADHD (Loo et al. 2013). These differences are unlikely to be due to EO or EC condition differences, as both positive and negative T:B findings have been reported in comparison studies (Lansbergen et al. 2011; Liechti et al. 2013; Loo et al. 2013, 2009; Ogrim et al. 2012; van Dongen-Boomsma et al. 2010; Woltering et al. 2012). A recent meta-analysis, conducted on studies using EO data, demonstrated that the reported T:B effect size showed a strong relationship with year of publication, declining over time (Arns et al. 2013). Arns et al. suggest this might be due to testing context differences between studies, the trend for reduced sleep duration in children across years, or sample differences.

The consistency of delta and beta differences in ADHD is more limited. Enhanced delta activity has been reported in children with ADHD (Barry et al. 2010; Bresnahan et al. 1999; Nazari et al. 2011; Swartwood et al. 2003), but may reflect a maturational lag and is also not consistently replicated in children (Clarke et al. 2002c, 2003b; Liechti et al. 2013), or adults (Koehler et al. 2009; Liechti et al. 2013).

Beta activity findings are conflicting in both EO and EC data, with a meta-analysis and other studies of children, adolescents and adults reporting attenuation (Barry et al. 2010; Bresnahan et al. 1999; Clarke et al. 2006; Shi et al. 2012; Snyder and Hall 2006); while other studies report enhancement in ADHD adults compared to children (Poil et al. 2014), enhancement in a subset of children who have high hyperactivive/impulsive symptoms (Clarke et al. 2001b, 2007), or in specific narrow-band beta frequency ranges (Loo et al. 2009). However, many studies fail to observe case–control differences in beta activity, in either children or adults (Buyck and Wiersema 2014; Koehler et al. 2009; Lansbergen et al. 2011; Liechti et al. 2013; Loo et al. 2013; Nazari et al. 2011; Ogrim et al. 2012; van Dongen-Boomsma et al. 2010; Woltering et al. 2012).

The inconsistencies in reported case–control differences contrast with spectral EEG’s robust sensitivity to age and maturational lag (Bresnahan et al. 1999; Liechti et al. 2013; Loo et al. 2013; Ogrim et al. 2012; Poil et al. 2014; Snyder and Hall 2006) and could reflect factors such as decreasing ADHD symptoms with age (Snyder and Hall 2006), ADHD subtype (Buyck and Wiersema 2014; Clarke et al. 2001a; Loo et al. 2013, 2010), medication (Clarke et al. 2003a, 2002a; Loo et al. 2004), and co-occurring symptoms of depression or disruptive behaviours (Clarke et al. 2002b; Loo et al. 2013). Few studies have directly explored the potential effects of IQ on EEG power in ADHD with most studies using samples with normal range or matched IQs, despite lower IQ commonly being associated with ADHD. One study on 40 children with ADHD reported EEG power to be similar in subgroups of children with both high and low IQ (Clarke et al. 2006), while (Chabot and Serfontein 1996) reported that, although there were ADHD-associated differences in spectral EEG for both low and high IQ groups, low IQ did contribute to generalised EEG differences in terms of greater asymmetry, and reduced alpha and/or theta power. This suggests that lower IQ, while not being the dominant cause of spectral profile differences seen in ADHD, may contribute to group differences in studies where the ADHD sample show typical lower mean IQ scores and where IQ is not otherwise controlled. Studies should therefore attempt to examine the influence of IQ on results, by comparing results with and without controlling for the effects of IQ on their data.

Other possible explanations of the inconsistencies observed between the studies could be related to differences in recording context (i.e., when recordings are conducted in relation to the start or end of a recording session or other experimental demands), which might influence the level of arousal in participants. Arousal may be more variable in ADHD and can affect symptom severity and performance (Sergeant 2005; Van der Meere 2002), and may therefore vary throughout an experimental record session. For example, rest-to-task comparisons show prominent EEG power differences (Loo et al. 2013; Nazari et al. 2011; Ogrim et al. 2012), while Koehler et al. (2009) report reduced beta and T:B differences between two resting state recordings completed at the beginning and end of the Eriksen Flanker Task.

We therefore hypothesised that differences in recording context, such as whether recordings are made at the start or end of an experimental session, may alter spectral EEG case–control differences, as the novelty of the testing environment declines with time, especially over longer recording durations. This study investigated if spectral power and global field synchronization (GFS) varies between ADHD and control groups in conventional spectral bands (delta, theta, alpha, beta) and in theta/beta ratio between recordings made at the beginning and end of a 1.5 h cognitive-EEG testing session. As a further post hoc analysis, we additionally examined whether IQ influences any ADHD-control differences that emerge.

## Methods and Materials

### Sample

ADHD and control participants who had taken part in our previous research (Chen et al. 2008; Kuntsi et al. 2010), were invited to take part in this follow-up study. In the initial study, ADHD participants aged between 6 and 17 were recruited from specialist clinics in the UK from among those who had a clinical diagnosis of DSM-IV combined subtype ADHD during childhood, as determined by a paediatrician or child psychiatrist. The control group were recruited from primary (ages 6–11 years) and secondary (ages 12–18 years) schools in the UK. At follow-up in this study, participants were aged between 13 and 25, and for this investigation ADHD participants were re-assessed and only those who continued to meet DSM-IV criteria for any ADHD subtype in adolescence/early adulthood were included in current analyses. All participants were of European Caucasian decent.

For both groups, the exclusion criteria, as defined by those used in the initial investigation, were IQ < 70, autism, epilepsy, learning difficulties, brain disorders and any genetic or medical disorder associated with externalising behaviours that might mimic ADHD. Written informed consent was obtained and the study was approved by the London-Surrey Borders Research Ethics Committee (NRES 09/H0806/58).

Six ADHD participants were excluded from the analysis (because of unusable EEG data (4) and <20 acceptable EEG segments (2)). Two control participants were excluded, as they met ADHD criteria based on parent report; and one further control participant had <20 acceptable EEG segments. The final sample consisted of 76 ADHD participants and 85 controls. The ADHD and control groups did not differ in age (ADHD: mean = 18.70, SD = 2.91; Control: mean = 18.29, SD = 1.76; t = −1.362, df = 181, *p* > 0.5), but differed significantly in full-scale IQ (ADHD: mean = 98.44, SD = 14.27; Control: mean = 111.67, SD = 12.86; t = −6.547, df = 181, *p* < 0.001) and in gender distribution (ADHD: 89 % male; Control: 99 % male; χ^2^(1, *n* = 183) = 4.75, *p* = 0.03).

### Procedure

Participants attended a single research session for clinical interviews and cognitive-EEG assessments, as part of a larger study. A 48-hour ADHD medication-free period was required before the research session. Two 3-minute eyes-open resting state conditions were administered at the beginning and end of an extended 1.5 h cognitive-EEG test battery. Participants were requested to remain still, and keep their eyes on a fixed point in front of them for the duration of the recording.

### Measures

#### ADHD diagnosis

Childhood ADHD was initially assessed using the Parental Account of Childhood symptoms (PACS) (Chen et al. 2008; Taylor et al. 1986a, 1986b), a semi-structured, standardised, investigator interview with high inter-rater reliability (Taylor et al. 1986a). During follow-up, ADHD status was confirmed using parental ratings of the Diagnostic Interview for ADHD in Adults (DIVA) (Kooij and Francken 2007) and the Barkley’s Functional Impairment Scale (BFIS) (Barkley and Murphy 2006). A research diagnosis of ADHD was made if participants scored ≥6 on the DIVA for either inattention or hyperactivity/impulsivity scales, and ≥2 positive scores on ≥2 areas of impairments on the BFIS, based on DSM-IV criteria. Six participants were excluded from the sample, as they had missing parent ratings of clinical impairment and their current ADHD status could therefore not be determined.

#### IQ

The vocabulary and block design subtests of the Wechsler Abbreviated Scale of Intelligence Fourth Edition (WASI-IV) (Wechsler 1999) were administered to all participants to derive an estimate of IQ.

#### EEG recording and Analysis

Two 3-minute fixed-gaze eyes-open resting conditions were carried out, at the beginning and end of a 1.5 h recording session. Participants completed three event related potential (ERP) paradigms between resting state recordings, administered in a fixed order (Continuous Performance Task (Doehnert et al. 2008); Eriksen Flanker Task (Albrecht et al. 2009); and the Fast Task (Andreou et al. 2007; Kuntsi et al. 2006)). The EEG was recorded from a 62 channel DC-coupled recording system (extended 10–20 montage), using a 500 Hz sampling-rate, impedances under 10 kΩ, and FCz as the reference electrode. The electro-oculograms (EOGs) were recorded from electrodes above and below the left eye and at the outer canthi.

The EEG data were analysed using Brain Vision Analyzer (2.0) (Brain Products, Germany). Raw EEG recordings were down-sampled to 256 Hz, re-referenced to the average of all electrodes, and digitally filtered using Butterworth band-pass filters (0.1–30 Hz, 24 dB/oct). Ocular artefacts were identified using independent component analysis (ICA) (Jung et al. 2000). All trials were also visually inspected for other subtle artefacts, and sections containing these were manually removed. Data with other artefacts exceeding ±100 μV in any channel or with a voltage step greater than 50 μV were rejected. Where an entire channel was removed due to technical problems or electrical noise, topographic spline interpolation was used to replace the channel.

The cleaned continuous EEG was then segmented into 2-second epochs and power spectra computed using the Fast Fourier Transform with a 10 % Hanning window. Epochs were averaged to create group means. Bands were defined as delta 0.5–3.5 Hz; theta 3.5–7.5 Hz; alpha 7.5–12 Hz; and beta 12–30 Hz. Topographic maps, t-maps and band-power graphs were generated from scalp recordings of power at all electrodes (see supplementary material S1–S3). In order to attempt to replicate findings from the majority of previous studies (Clarke et al. 2002c, 2003b; Koehler et al. 2009; Lansbergen et al. 2011; Loo et al. 2009, 2004; Loo and Smalley 2008; van Dongen-Boomsma et al. 2010), EEG power was averaged into three regions from individual scalp electrodes (frontal: Fz, F1, F2, F3, F4, F5, F6, F7, F8; central: Cz, C1, C2, C3, C4, C5, C6; parietal: Pz, P3, P4, P7, P8). For an additional comparison with more recent investigations (Buyck and Wiersema 2014; Liechti et al. 2013; Loo et al. 2013; Ogrim et al. 2012; Woltering et al. 2012) and to discount the effect of electrode selection, we also re-ran all analyses using only mid-line electrodes (Fz, Cz, Pz).

The observed absolute power within any given band is based upon the phase and amplitude of multiple EEG sources. When sources are phase-locked, they are synchronised, indicating they are simultaneously active within the brain. GFS (Koenig et al. 2001, 2005) is an index of phase synchrony at a given frequency. It provides an additional dimension beyond absolute power for understanding the global functional connectivity within these frequency bands, with the advantage of being a relative measure, which is not influenced by the choice of reference electrode. GFS provides a single score between 0 and 1, with zero indicating no synchrony between EEG sources, and 1 indicating all sources are in phase. GFS was computed for each 2-second epoch, averaged for each participant and then examined by frequency band.

### Statistical Analyses

An exploratory analysis on age effects was carried out by comparing power within each band between the younger (13–18 years old) and older (>18) subsets within each group. Based on this analysis (supplementary material S4), which indicated the older group to have reduced power in all bands, we included age, along with gender, as covariates in all analyses. In addition, all analyses were re-run with IQ as an additional covariate to examine empirically the effects of IQ on EEG power. Mean power was non-normally distributed and transformed using log for conventional frequency bands, and square root for theta/beta ratio. A repeated measures analysis of covariance (ANCOVA) was carried out in SPSS (version 21) within each band (delta, theta, alpha, beta), for both EEG power and GFS measurements, and within theta/beta ratio for EEG power only. Two within-subjects factors were included: time (start and the end of the testing session) and region (frontal, central, parietal or Fz, Cz, Pz electrodes); and one between-subject factor (group). Where necessary, to examine group differences at either time-1 or -2 individually, subsequent follow-up ANCOVAs were performed using only group and region factors. We focused both on p-values (*p* < 0.05 for significance, and *p* < 0.08 for a trend) and effect sizes (eta squared (η^2^)). Based on (Cohen’s 1988, p.283), estimates for η^2^, 0.0099 constitutes a small effect, 0.0588 a medium effect and 0.1379 a large effect.

## Results

### Group Differences

**Fig. 1:**
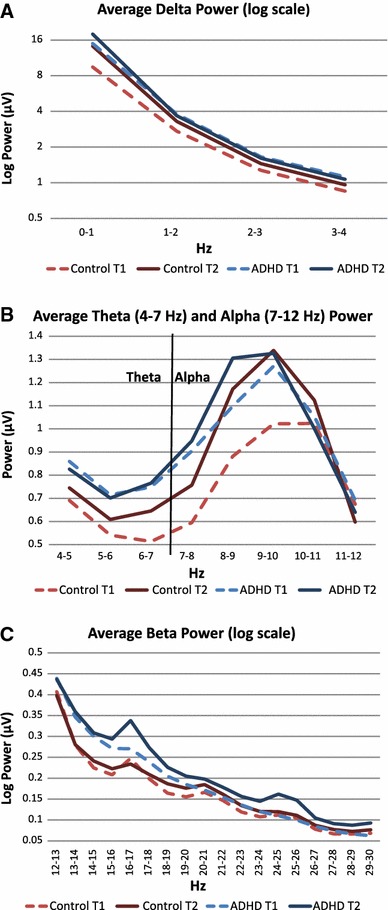
Average spectral mean EEG power across bands. Average spectral power in ADHD and controls groups at time-1 and time-2, by frequency band. *Plots* represent mean power across from frontal, central and parietal regions in the ranges of **a** delta (0.5–3.5 Hz); **b** theta (3.5–7.5 Hz) and alpha (7.5–12 Hz) and **c** beta 12–30 Hz

An ANCOVA indicated significantly higher delta power in the ADHD group, compared to controls (Table 1 and Fig. 1a). A post hoc analysis showed that the group means (Table 2) differed significantly for delta power at time-1 (F(1, 157) = 7.81, *p* = 0.01, η^2^ = 0.0437), but not at time-2 (F(1, 157) = 0.36, *p* = 0.55, η^2^ = 0.0022). For theta band (Table 1 and Fig. 1b), an effect of group at trend level was observed. Post-hoc analysis indicated that the ADHD group had significantly higher mean theta power than controls at time-1 (F(1, 157) = 6.46, *p* = 0.01, η^2^ = 0.0329) but not at time-2 (F(1, 157) = 0.94, *p* = 0.33, η^2^ = 0.0052). In the alpha band (Table 1 and Fig. 1b), no significant group differences emerged. For beta activity, we observed a main effect of group (Table 1 and Fig. 1c), with post hoc analysis indicating a significantly higher mean beta power in ADHD than control group at time-2 (F(1, 157) = 5.68, *p* = 0.018, η^2^ = 0.0318), but not at time-1 (F(1, 157) = 2.90, *p* = 0.09, η^2^ = 0.0154). All main effect group comparisons in conventional bands had small effect sizes. The main effect of group for theta/beta ratio was not significant, and had a minimal effect size.

**Table 1:** Significance values and effect sizes for ANCOVA factors and interactions, controlling for age and gender

	Delta	Theta	Alpha	Beta	T:B
Time					
F	0.618	2.402	1.907	0.286	0.013
*p*	0.433	0.123	0.169	0.594	0.910
η^2^	0.0038	0.0141	0.0113	0.0018	0.000
Region					
F	5.477	3.922	4.561	0.916	1.916
*p*	0.005*	0.021*	0.011*	0.401	0.149
η^2^	0.0317	0.0233	0.0275	0.0058	0.012
Group					
F	4.294	3.747	1.635	5.478	0.067
*p*	0.040*	0.055^a^	0.203	0.021*	0.796
η^2^	0.0245	0.0191	0.0095	0.0288	0.000
Group* region					
F	2.023	1.376	0.364	0.464	1.461
*p*	0.134	0.254	0.695	0.594	0.234
η^2^	0.0117	0.0082	0.0022	0.0029	0.009
Group* time					
F	3.479	3.717	0.832	0.582	3.112
*p*	0.064^a^	0.056^a^	0.363	0.447	0.080^a^
η^2^	0.0214	0.0218	0.0049	0.0036	0.019

Activity bands defined as: delta 0.5–3.4 Hz, theta 3.5–7.5 Hz, alpha 7.5–12 Hz, beta 12–30 Hz

* Denotes significant at *p* < 0.05

^a^Denotes trend level effect at *p* < 0.08. Effect size (η^2^); 0.0099 constitutes a small effect, 0.0588 a medium effect and 0.1379 a large effect

**Table 2:** Mean amplitude in µV and standard deviation (SD), prior to transformations, and with age and gender controlled for, in ADHD and control groups across frequency bands and theta/beta ratio at frontal, central and partial regions

	Delta µV (SD)	Theta µV (SD)	Alpha µV (SD)	Beta µV (SD)	T:B µV (SD)
Frontal Region					
T1					
Control	3.585 (0.29)	0.557 (0.04)	0.567 (0.06)	0.146 (0.01)	2.127 (0.06)
ADHD	2.431 (0.19)	0.724 (0.04)	0.703 (0.06)	0.173 (0.01)	2.189 (0.07)
T2					
Control	4.436 (0.29)	0.654 (0.04)	0.652 (0.05)	0.157 (0.01)	2.190 (0.06)
ADHD	3.308 (0.2)	0.765 (0.04)	0.781 (0.06)	0.202 (0.01)	2.108 (0.06)
Central Region					
T1					
Control	3.361 (0.2)	0.563 (0.05)	0.698 (0.08)	0.153 (0.01)	2.120 (0.06)
ADHD	2.181 (0.19)	0.752 (0.05)	0.916 (0.08)	0.170 (0.01)	2.206 (0.06)
T2					
Control	3.793 (0.21)	0.606 (0.05)	0.822 (0.09)	0.155 (0.01)	2.192 (0.06)
ADHD	2.542 (0.19)	0.732 (0.05)	0.950 (0.1)	0.200 (0.01)	2.154 (0.07)
Parietal Region					
T1					
Control	3.029 (0.2)	0.696 (0.07)	1.193 (0.15)	0.186 (0.01)	2.060 (0.06)
ADHD	2.982 (0.27)	0.986 (0.07)	1.459 (0.16)	0.214 (0.01)	2.185 (0.06)
T2					
Control	2.935 (0.2)	0.788 (0.07)	1.438 (0.16)	0.195 (0.01)	2.139 (0.06)
ADHD	4.378 (0.3)	0.952 (0.07)	1.492 (0.17)	0.241 (0.01)	2.106 (0.07)

Activity bands defined as: delta 0.5–3.5 Hz, theta 3.5–7.5 Hz, alpha 7.5–12 Hz, beta 12–30 Hz. Regions are average power from individual electrodes: frontal: Fz, F1, F2, F3, F4, F5, F6, F7, F8; central: Cz, C1, C2, C3, C4, C5, C6; parietal: Pz, P3, P4, P7, P8

### Group by Time Interactions

Group by time interactions emerged at trend level for delta and theta bands, and were not significant for alpha and beta bands. Effect sizes were small for delta and theta, and minimal in alpha and beta bands (Table 1). A trend-level group by time interaction was detected for theta/beta ratio, which had a small effect size. Post-hoc analysis did not show group differences in theta/beta ratio at either time-1 (F(1, 157) = 1.08, *p* = 0.30, η^2^ = 0.0066) or 2 (F(1, 157) = 0.350, *p* = 0.56, η^2^ = 0.0021).

### Time

The main effects of time, independent of group, were not significant in any of the four spectral bands, or for theta/beta ratio (Table 1). Theta and alpha bands had a small effect size; in delta, beta and theta/beta ratio the effect size was minimal.

### Controlling for IQ

To examine the effect of IQ on EEG spectral power, all analyses were re-run including IQ as an additional covariate. This altered the significance of several comparisons (Table 3). Specifically, group differences in delta and beta bands weakened to trend level and or non-significance respectively, and the prior trend in theta became non-significant. However, in all three cases a small effect size was maintained. When controlling for IQ, group differences in alpha and theta/beta ratio remained non-significant, but the group by time interactions emerged as significant, although with small effect sizes, for delta and theta bands. Post-hoc analysis indicated that these significant group by time interactions in delta and theta bands were driven by significant group differences at time-1 (delta: F(1, 157) = 7.32, *p* = 0.01, η^2^ = 0.0412; theta: F(1, 157) = 5.07, *p* = 0.03, η^2^ = 0.0255), which were not present at time-2 (delta: F(1, 157) = 0.09, *p* = 0.76, η^2^ = 0.0006; theta: F(1, 157) = 0.26, *p* = 0.61, η^2^ = 0.0015). The trend level group by time interaction for theta/beta ratio became non-significant when controlling for IQ.

**Table 3:** Significance values and effect sizes for ANCOVA factors and interactions, controlling for age, gender and IQ

	Delta	Theta	Alpha	Beta	T:B
Time					
F	0.006	0.393	0.616	0.188	0.029
*p*	0.94	0.531	0.434	0.665	0.865
η^2^	0.0000	0.0023	0.0037	0.0012	0.0001
Region					
F	2.874	1.735	2.638	0.245	1.008
*p*	0.058 ^a^	0.178	0.073 ^a^	0.783	0.366
η^2^	0.0170	0.0105	0.0162	0.0016	0.0118
Group					
F	3.373	2.321	1.68	2.483	0.179
*p*	0.068 ^a^	0.13	0.197	0.117	0.673
η^2^	0.0195	0.0122	0.0098	0.0135	0.0004
Group* region					
F	1.214	0.673	0.277	0.113	2.148
*p*	0.298	0.511	0.758	0.893	0.145
η^2^	0.0072	0.0041	0.0017	0.0007	0.0090
Group* time					
F	4.178	4.553	1.039	0.001	0.939
*p*	0.043*	0.034*	0.31	0.997	0.392
η^2^	0.0257	0.0268	0.0062	0.0000	0.0194

Activity bands defined as: delta 0.5–3.5 Hz, theta 3.5–7.5 Hz, alpha 7.5–12 Hz, beta 12–30 Hz

* Denotes significant at *p* < 0.05, unadjusted

^a^denotes trend level effect at *p* < 0.08. Underlined values indicated those which changed between significant/non-significant when including IQ as a covariate. Effect size (η^2^); 0.0099 constitutes a small effect, 0.0588 a medium effect and 0.1379 a large effect

### Analysis Using Mid-Line Electrodes

Re-running analysis based on mid-line electrodes, compared to frontal, central and parietal regions yielded similar results, with some exceptions. Without controlling for IQ, the reported group by time trend for theta became significant (F(1,157) = 6.92, *p* = 0.01, η^2^ = 0.0274), and group differences for beta became non-significant, although the small effect size remained (F(1,157) = 4.19, *p* = 0.11, η^2^ = 0.0141). The significant difference in region for delta also became non-significant (F(1,157) = 2.08, *p* = 0.13, η^2^ = 0.0009). When IQ was controlled for, an additional time by group interaction in alpha was detected (F(1,157) = 2.21, *p* = 0.01, η^2^ = 0.0132), and the trend for the group by time interaction in delta became significant (F(1,157) = 5.03, *p* = 0.03, η^2^ = 0.026). Full results are reported in supplementary material (S5, S6 & S7).

### Global Field Synchronisation

Mean GFS scores (Table 4) did not differ between groups at either time point, or between time-1 and 2 in any band (supplementary material S8). The addition of IQ as an additional covariate did not alter results. Age, as a covariate, had a significant relationship to GFS scores in all bands (supplementary material S8). We ran additional correlations to investigate the age effect further (supplementary material S9), which showed that age was positively correlated with GFS scores in the majority of bands, except time-1 beta and time-2 theta (which were at trend level) and time-2 beta (which was non-significant).

**Table 4:** Global Field Synchronisation Scores

	Delta GFS (SD)	Theta GFS (SD)	Alpha GFS (SD)	Beta GFS (SD)
ADHD				
T1	0.46 (0.04)	0.44 (0.03)	0.47 (0.04)	0.45 (0.04)
T2	0.46 (0.04)	0.44 (0.03)	0.46 (0.03)	0.46 (0.04)
Control				
T1	0.45 (0.03)	0.43 (0.03)	0.47 (0.04)	0.44 (0.03)
T2	0.45 (0.04)	0.44 (0.04)	0.47 (0.04)	0.46 (0.05)

Activity bands defined as: delta 0.5–3.5 Hz, theta 3.5–7.5 Hz, alpha 7.5–12 Hz, beta 12–30 Hz

## Discussion

We report evidence for the influence of time-context effects on whether EEG spectral power differences emerge between participants with ADHD and controls. At the start of the recording session, delta as well as theta power was elevated in the ADHD group, while at the end of the recording session ADHD was linked only to elevated activity in the beta band. In addition, trend level group by time interactions in delta and theta bands, which became significant when controlling for IQ, in conjunction with graphed power (Fig. 1), indicate that activity in delta and theta bands was consistently high in the ADHD group, whereas the control group showed time-related changes. This finding supports theories of hypoarousal in ADHD (Weinberg and Brumback 1990), which would argue for persistent under-activation in ADHD at both time points. Yet, work based on combining EEG with skin conductance recordings has associated increased alpha, instead of increased theta or T:B ratios, with hypoarousal (Barry et al. 2009), rendering this interpretation somewhat tentative. We did not find evidence for atypical T:B ratio or alpha activity in the current sample of adolescents and young adults with ADHD. In this investigation, as expected, IQ was significantly lower in the ADHD group (Kuntsi et al. 2004; Wood et al. 2011). Controlling for IQ slightly altered the pattern of results, reducing group main effects, but strengthening group by time interactions for delta and theta bands. This is consistent with the small but generalised effect of IQ on EEG power as reported by (Chabot and Serfontein 1996), and illustrates that IQ can influence EEG results and should be empirically explored in studies on populations with lower IQ scores, such as individuals with ADHD.

Our findings provide no support for the initial hypothesis that under-arousal (as reflected by increased theta or alpha) among individuals with ADHD is more likely to be observed in a familiar setting and is reduced in a novel testing environment. Instead they show that under-activation, as indexed by delta and theta activity, may be present throughout testing. However, other explanations could include the influence of the preceding tasks at time-2 which may have influenced arousal. Changes over time could be examined directly in future studies by conducting short resting-state recordings throughout the EEG session to explore whether activation changes in a linear fashion over time, or alternatively, changes in relation to other tasks the participants are asked to complete during the recording session.

We did not detect any significant differences in alpha band activity in this study. As alpha has been negatively correlated with arousal, differences were expected (Barry et al. 2009). The spectra (Fig. 1b) are suggestive of group and time differences in the lower alpha band, particularly around 8-10 Hz, but less so at higher frequencies. It is possible that potential group differences were obscured here by averaging activity across full-band ranges, although other groups have found alpha power increases in adults with ADHD using the full alpha band (Koehler et al. 2009). Future analyses could examine time–frequency data at finer resolution to provide more power to detect group differences.

This study also did not replicate elevated T:B in the ADHD group at either time point, despite a sample size of 76 participants with persistent ADHD and 85 controls. This finding is at odds with older studies (Barry et al. 2010; Bresnahan et al. 1999; Clarke et al. 2001b, 2003b; Koehler et al. 2009; Lansbergen et al. 2011; Shi et al. 2012; Snyder and Hall 2006; Woltering et al. 2012), but consistent with several more recent investigations (Buyck and Wiersema 2014; Liechti et al. 2013; Loo et al. 2009; Ogrim et al. 2012; Poil et al. 2014; Ponomarev et al. 2014; Skirrow et al. paper under review; Swartwood et al. 2003; van Dongen-Boomsma et al. 2010), although Buyck and Wiersema showed subtype differences, with adult inattentive-type ADHD having lower T:B than the combined-type ADHD or controls. This questions the reliability of spectral analysis of resting state data to discriminate ADHD adolescents and young adults from controls, particularly as expected maturational effects are observed in this data (supplementary material S4), and that this sample also shows typical ADHD associated impairments in ERP and spectral EEG comparisons in data from a Continuous Performance Task recorded between the two resting state recordings as reported here (Cheung et al. under review).

Our additional analyses indicated some effects relating to the selection of electrodes. Focusing on mid-line electrodes (Fz, Cz, Pz) improved power to detect differences in theta and alpha bands. However, the opposite was observed for the beta band. T-maps indicated that group differences in beta activity at time-2 were detected broadly across multiple electrodes, while differences between time-1 and -2 in both the ADHD and control groups were greatest at fronto-lateral regions, including F7 and F8, which were included in our analysis as part of the frontal electrode region (supplementary material S2 & S3). Therefore, regions of electrodes which were more widely distributed across the scalp may have been more sensitive to beta differences, although were seemingly less sensitive to theta or alpha differences. This suggests that different methods of electrode selection may alter results, and as methods appear to have alternate sensitivity to detection of theta or beta power, may contribute to the declining replication of T:B differences in ADHD (Arns et al. 2013); particularly as most recent studies have favoured analysis of mid-line electrodes (Buyck and Wiersema 2014; Liechti et al. 2013; Loo et al. 2013; Ogrim et al. 2012; Woltering et al. 2012). Nonetheless, this cannot be the only factor influencing results, as we were unable to replicate T:B differences for ADHD using either method, similar to Liechti et al. (2013).

Differences in our results depending on electrode selection suggest that the standardisation of methods is important to ensure studies are comparable. As the maximal power of each band varies in location, adoption of new data-driven methods, as opposed to methods based on convention, may yield more reliable case–control differences. This might be achieved through analysis of all possible channel comparisons with appropriate multiple testing corrections (Poil et al. 2014; Woltering et al. 2012), by only selecting the channel where power is maximal based on topographic maps, similar to methods employed in ERP studies, or through the use of Independent Component Analysis to extract estimates of band power from multiple sources simultaneously (Ponomarev et al. 2014).

No group or condition differences in GFS scores were observed. Mean GFS scores were lower than in other published papers in adult and older adult populations, which are reported to be around approximately 0.5-0.55 (Kikuchi et al. 2007; Koenig et al. 2005; Ma et al. 2014; Pugnetti et al. 2010). In our study, age had a significant effect on GFS scores in most bands, in contrast to group status or condition variables. Significant correlations with age indicated that GFS score increased with age, which could suggest lower phase synchronization in younger participants at earlier stages of cortical maturation, compared to adult samples. This GFS increase parallels the spectral power reduction with maturation which also extended across bands, and demonstrates that GFS is sensitive to additional aspects of maturation. The finding is also in line with other studies that identified higher GFS scores in adults compared to children during a working memory paradigm (Michels et al. 2012), and with the maturational increases reported for alpha GFS (Koenig and Pascual-Marqui 2009).

In conclusion, we demonstrate that ADHD-control differences on EEG spectral power varied with recording time within a single recording session and with the frequency bands, although the modest effect sizes indicated that case–control discrimination was insufficient for diagnostic applications at both recording times. Our findings suggest that recording delta and theta activity during resting state at the start of recording sessions, where case–controls differences are likely to be highest as a product of persistent hypoarousal in ADHD, offers methodological advantages. In contrast, as beta activity increases over time in the ADHD group compared to controls, case–control differences in beta are likely to become more prominent in resting-state data recorded at the end of recording sessions. Our post hoc comparisons also indicate that data from electrode regions, compared to midline electrodes, may be more sensitive to differences in beta band activity, but not activity in delta and theta bands. Overall, this suggests that research design may be optimised for ADHD case–control differences at specific spectral frequency ranges. Such optimisation is likely to also apply to subtyping/clustering and treatment prediction based on resting EEG. However, we also highlight the need for studies to adopt consistent methodologies in the recording of data and to account for other factors such as electrode selection in their analyses. We also demonstrated that IQ has a small but significant influence on observed differences, and therefore should be taken into account in future investigations. Equally, we provide further evidence showing that age correlates with both EEG power and GFS scores, and should continue to be accounted for in future studies. While overall our findings of case-control differences in specific EEG power bands supports the view of arousal dysregulation in ADHD, our findings also demonstrate the challenges associated with the analysis and interpretation of resting state data in ADHD. Therefore we suggest that, until the factors that can influence the pattern of results are better understood, the use of resting-state band power as an associated feature supporting diagnosis for ADHD in adolescents and young adults is premature.

## Electronic supplementary material

Below is the link to the electronic supplementary material.

Supplementary material 1 (DOCX 916 kb)
