# Supplementary material for: A Matter of Time: The Influence of Recording Context on EEG Spectral Power in Adolescents and Young Adults with ADHD
Source: Brain Topogr. 2014 Sep 9;28(4):580–90. doi: 10.1007/s10548-014-0395-1 (PMC4475242; doi:10.1007/s10548-014-0395-1)
Supplement: Supplementary file 1 — Supplementary material 1 (DOCX 916 kb) [file 10548_2014_395_MOESM1_ESM.docx]

# S1. Topographic maps showing scalp recorded power density in delta, theta, alpha, and beta bands

|  | **Time 1** | | | | **Time 2** | | | |
| --- | --- | --- | --- | --- | --- | --- | --- | --- |
|  | **DELTA** | **THETA** | **ALPHA** | **BETA** | **DELTA** | **THETA** | **ALPHA** | **BETA** |
| **CONTROL** | 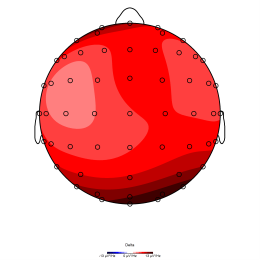 | 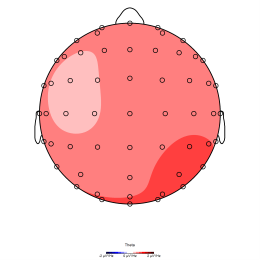 | 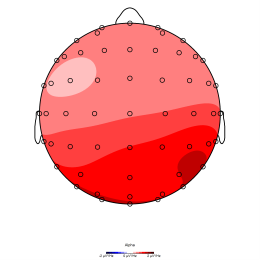 | 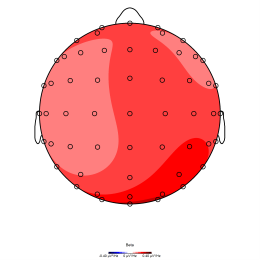 | 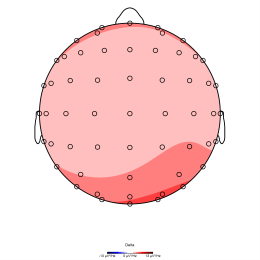 | 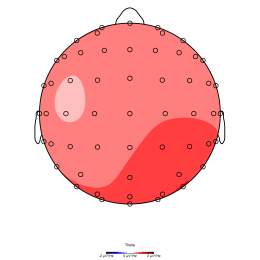 | 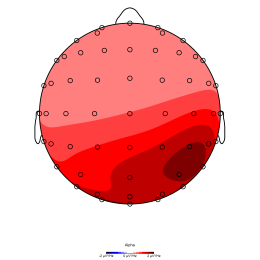 | 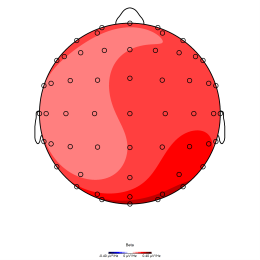 |
| **ADHD** | 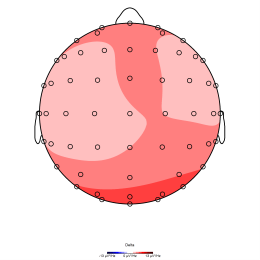 | 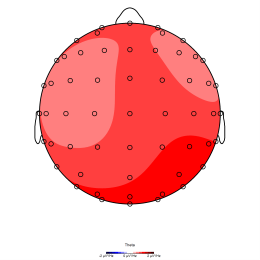 | 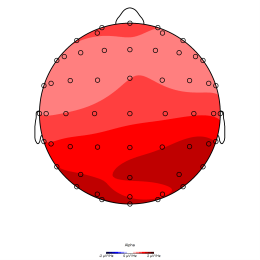 | 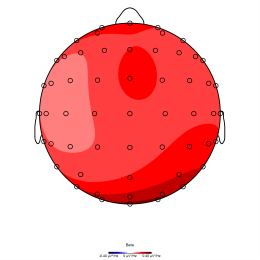 | 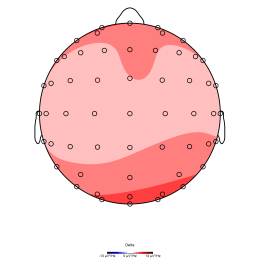 | 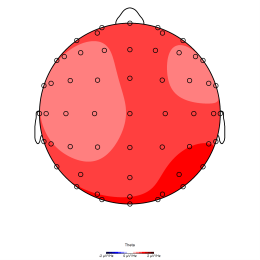 | 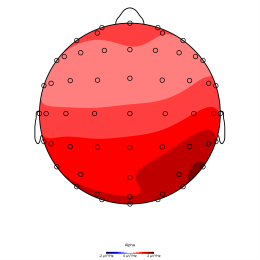 | 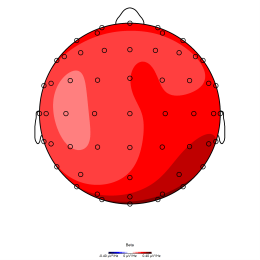 |
| **SCALES** | 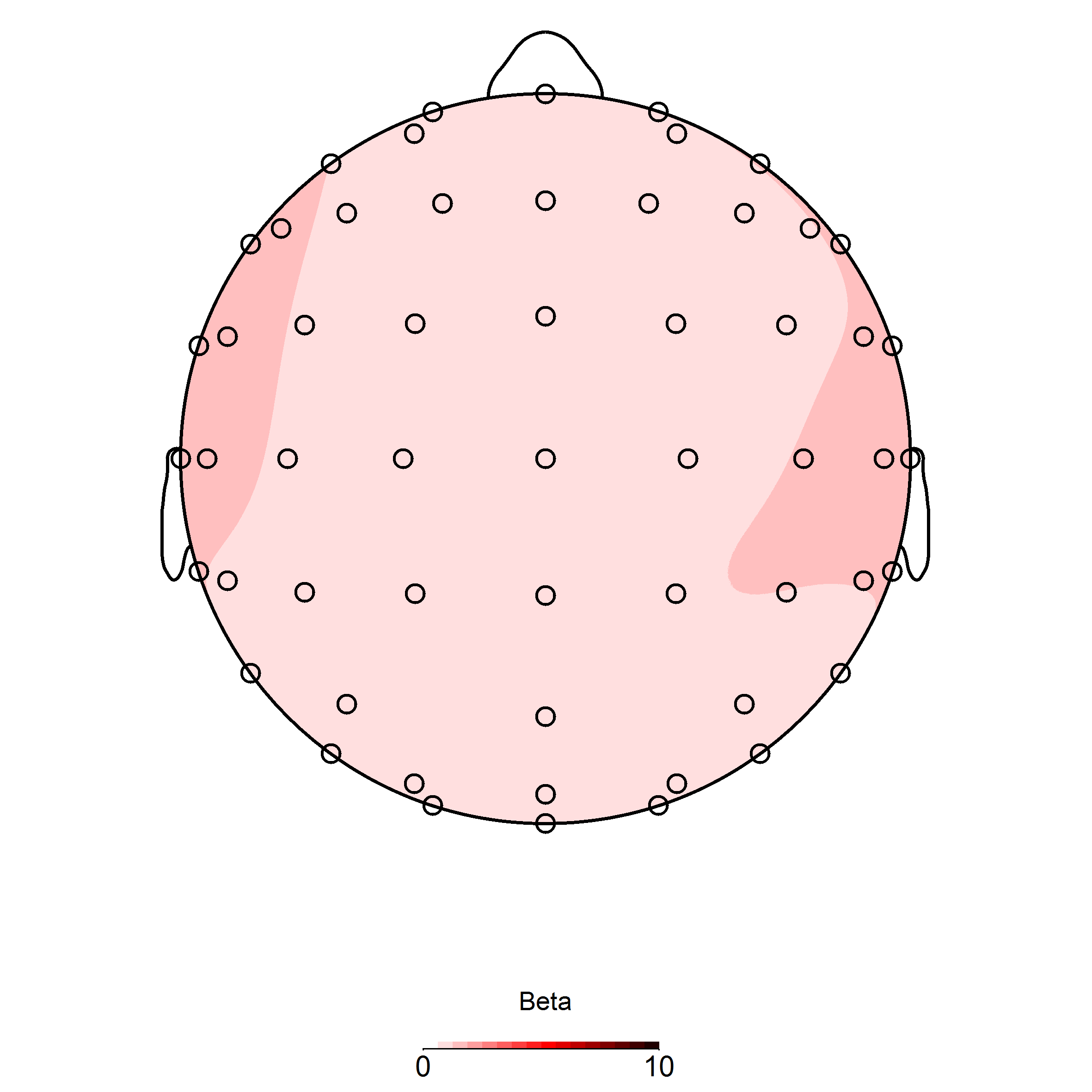 | | | | 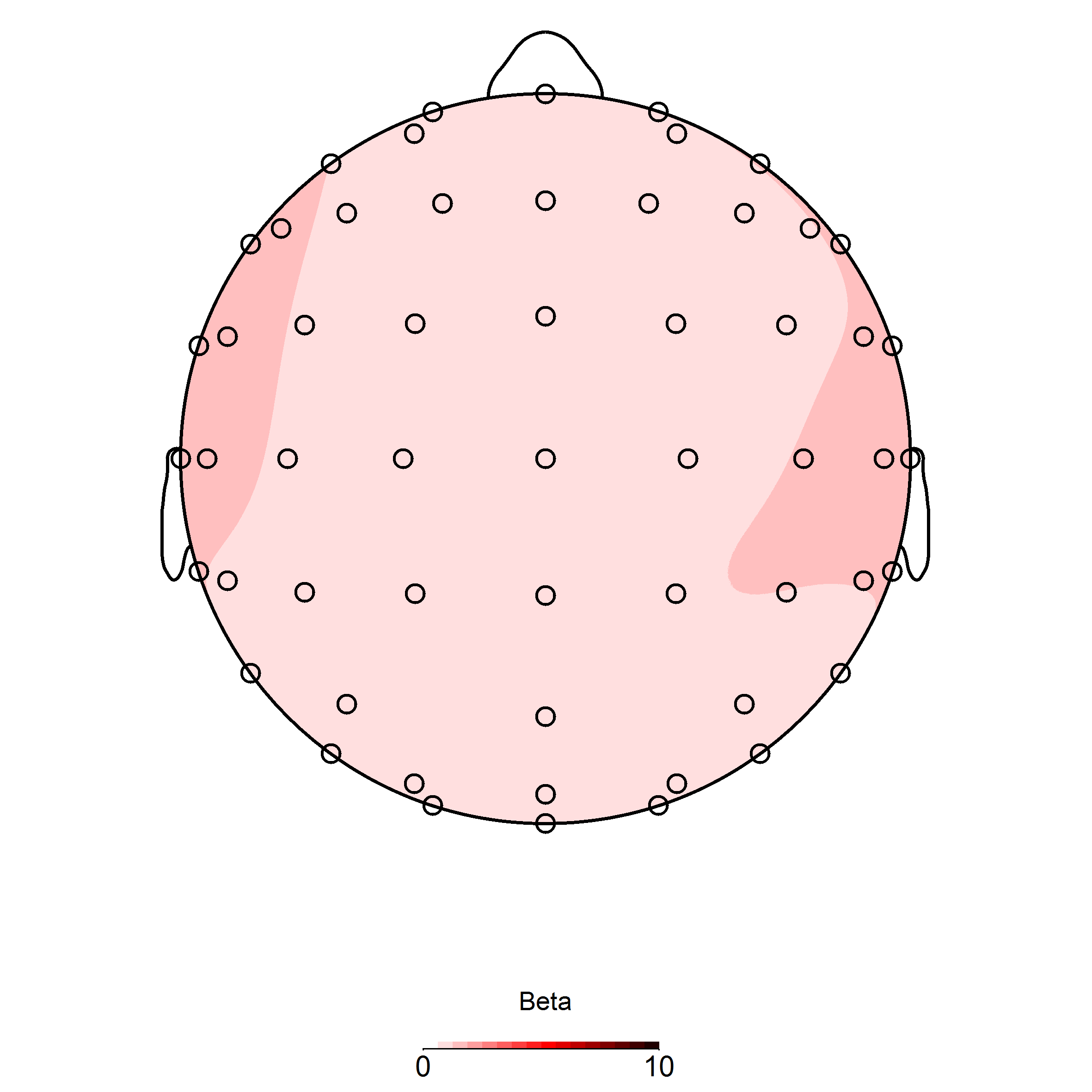 | | | |
|  | 0 to 13µV^2^/Hz | 0 to 2µV^2^/Hz | 0 to 2µV^2^/Hz | 0 to 0.4µV^2^/Hz | 0 to 13µV^2^/Hz | 0 to 2µV^2^/Hz | 0 to 2µV^2^/Hz | 0 to 0.4µV^2^/Hz |

# S2. T-maps showing time-1 activity relative to time-2 activity, across frequency bands and group status

|  | **(T2>T1)** | | | |
| --- | --- | --- | --- | --- |
|  | **DELTA** | **THETA** | **ALPHA** | **BETA** |
| **CONTROL** | 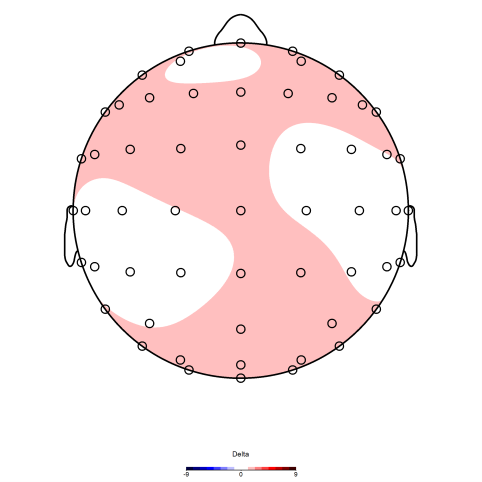 | 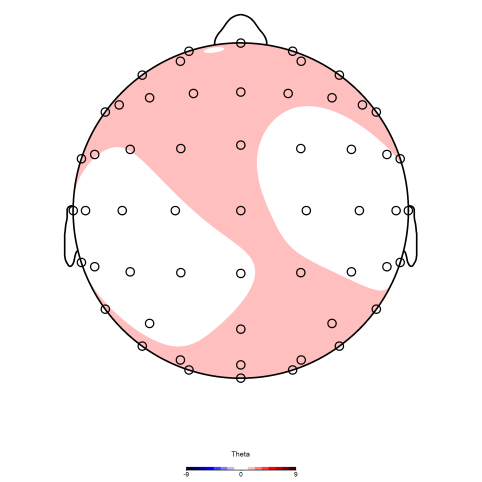 | 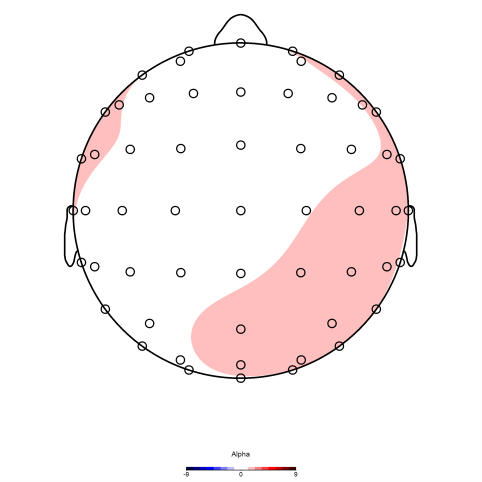 | 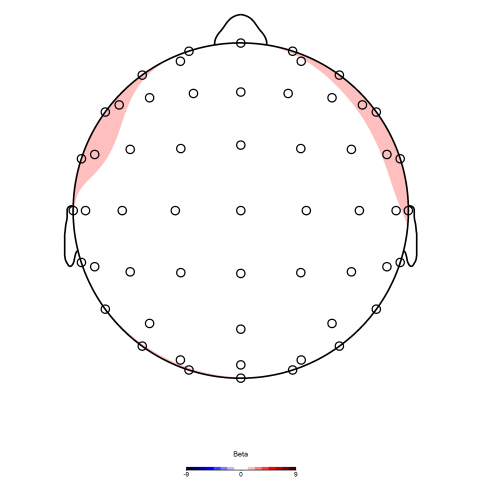 |
| **ADHD** | 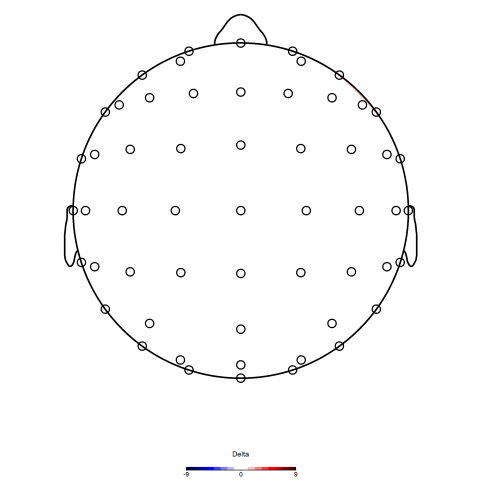 | 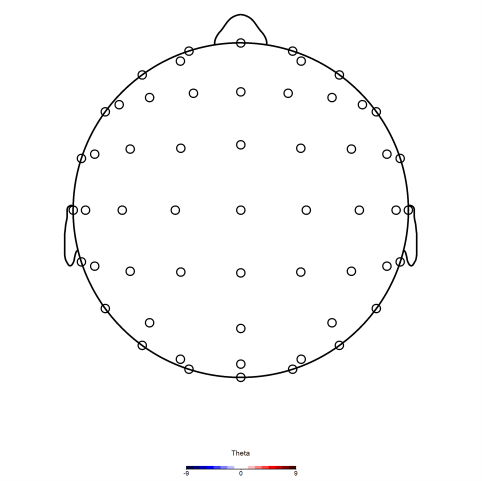 | 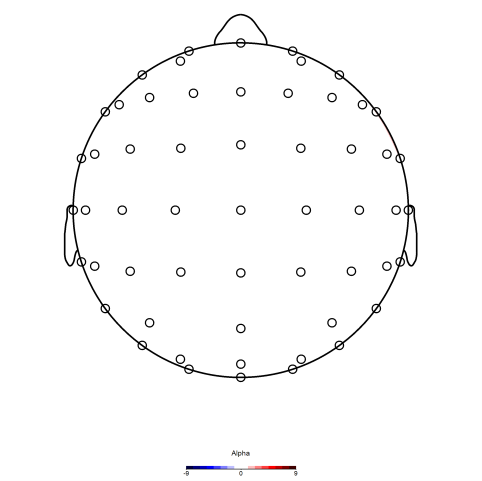 | 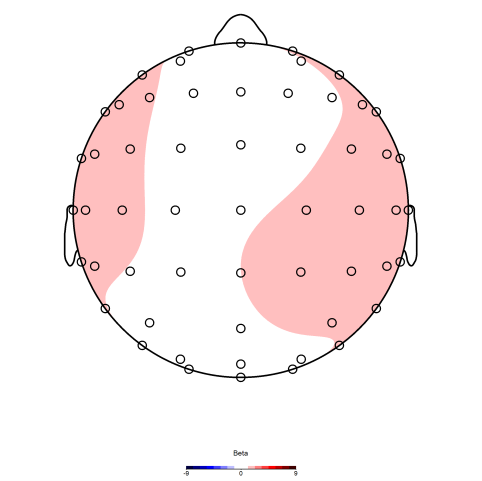 |
| **T-SCORE** | -9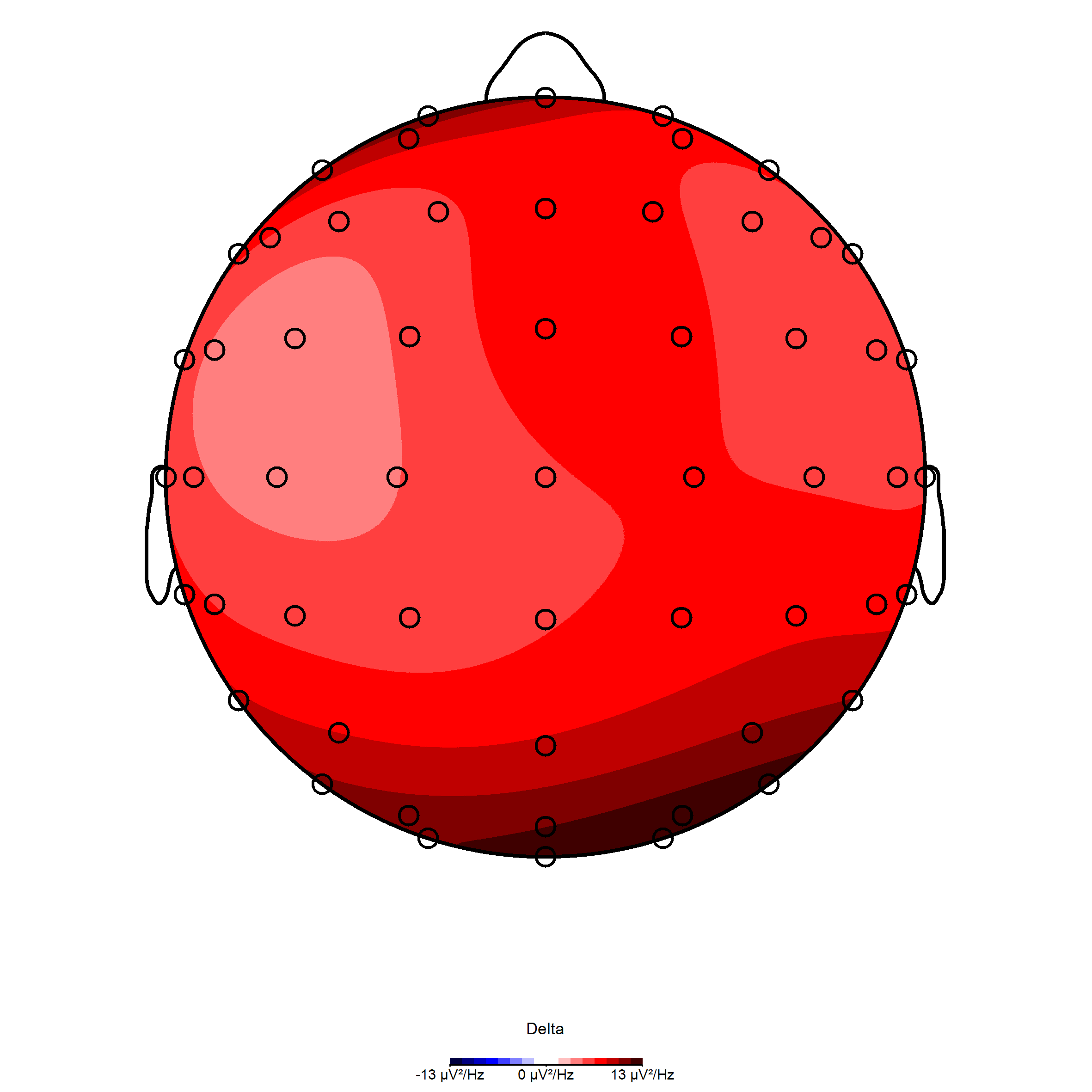9 | | | |

# S3. T-maps showing ADHD activity relative to control activity, across frequency bands and recording time

|  | **(ADHD>Control)** | | | |
| --- | --- | --- | --- | --- |
|  | **DELTA** | **THETA** | **ALPHA** | **BETA** |
| **Time 1** | 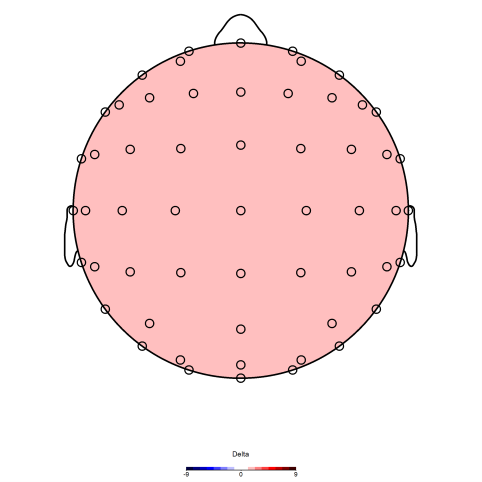 | 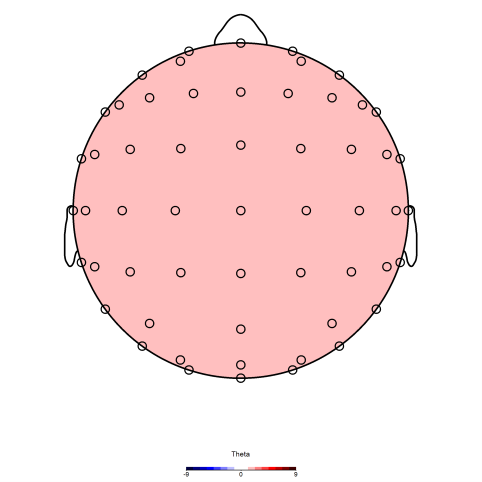 | 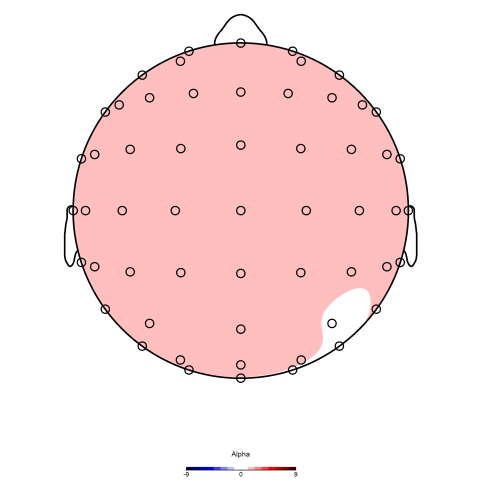 | 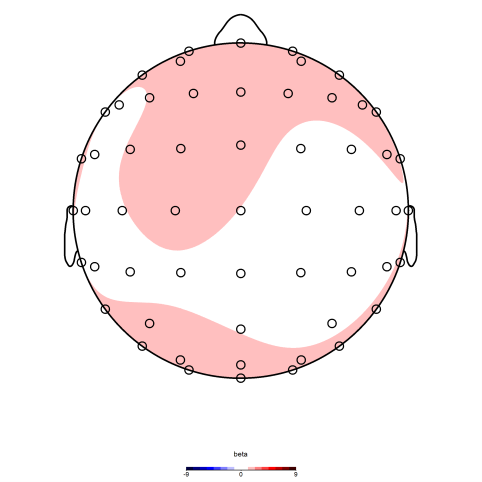 |
| **Time 2** | 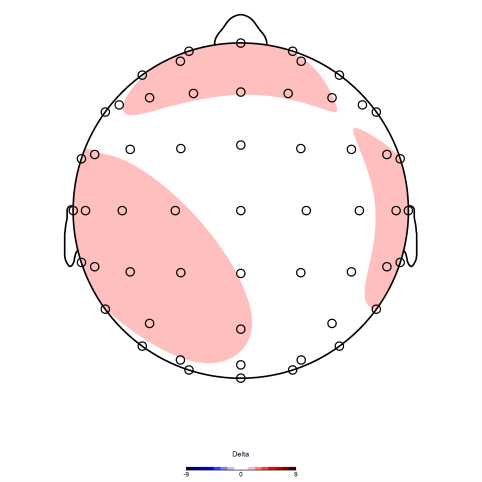 | 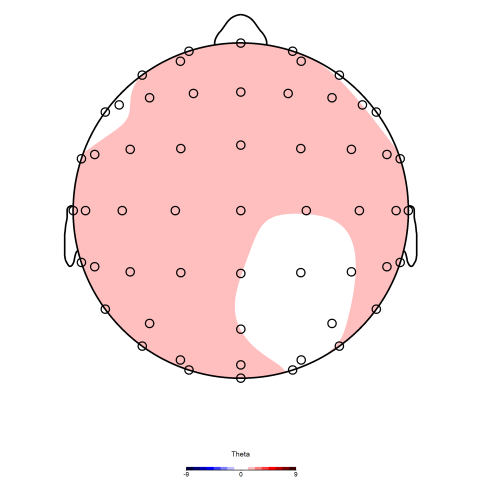 | 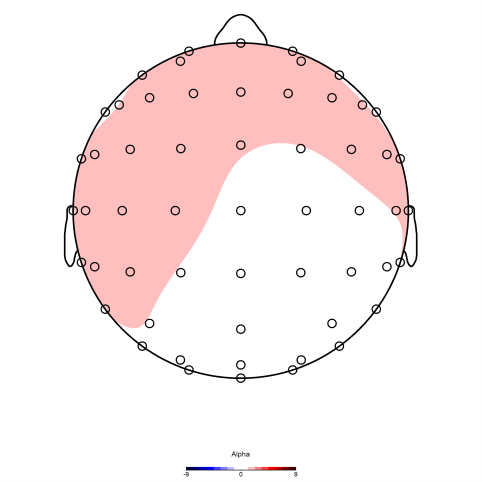 | 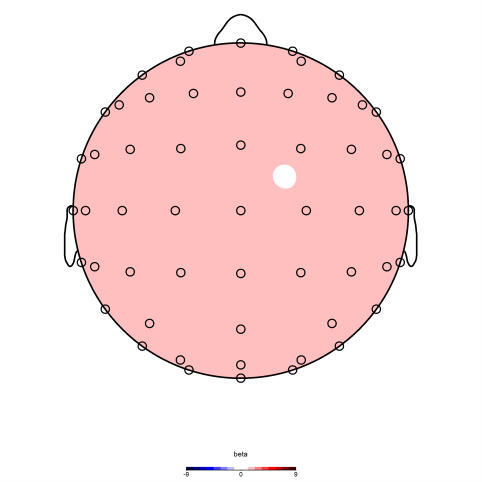 |
| **T-SCORE** | **-9**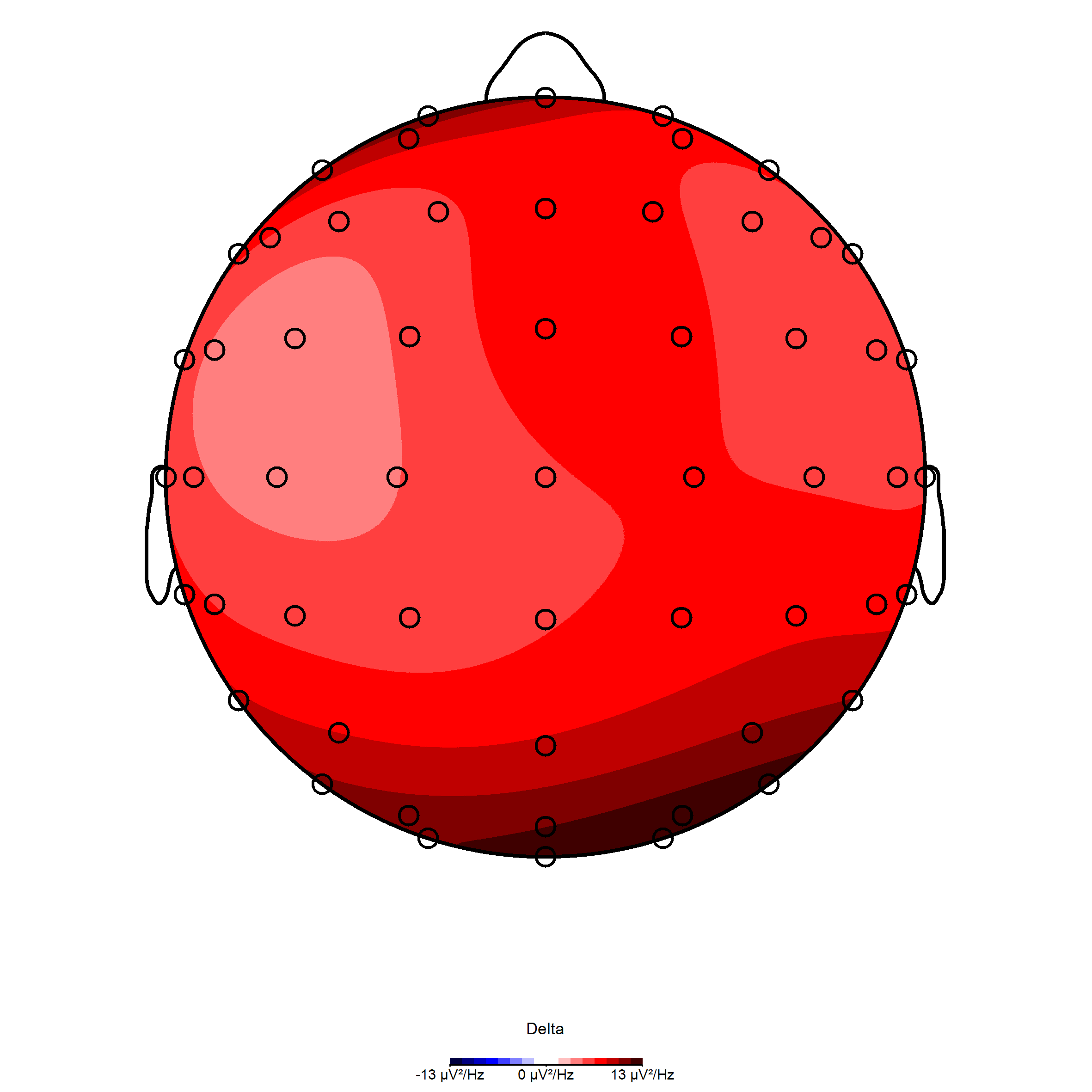**9** | | | |

# S4. Comparison of adolescents aged 12-18 and adults 18+ within ADHD and control samples.

|  |  |  | **ADHD** |  | **test statistic**  **age-comparison** | | **Control** | | **test statistic**  **age-comparison** | |
| --- | --- | --- | --- | --- | --- | --- | --- | --- | --- | --- |
|  | **Age** |  | **12-18** | **18+** |  |  | **12-18** | **18+** |  |  |
|  | **n** |  | 38 | 38 |  |  | 53 | 32 |  |  |
|  |  | **Region** | **µV (SD)** | **µV (SD)** | **F** | **p** | **µV (SD)** | **µV (SD)** | **F** | **p** |
| **Delta** | Time 1 | Frontal | 4.5 (2.6) | 2.15 (0.92) | 7.939 | 0.006* | 2.8 (1.41) | 1.91 (1.06) | 10.129 | 0.002* |
|  |  | Central | 4.12 (2.69) | 1.87 (1.02) |  |  | 2.57 (1.42) | 1.62 (0.75) |  |  |
|  |  | Parietal | 5.96 (4.06) | 2.79 (1.55) |  |  | 3.52 (1.93) | 2.24 (1.26) |  |  |
|  | Time 2 | Frontal | 4.44 (2.55) | 3.04 (1.46) |  |  | 3.69 (1.78) | 2.79 (1.23) |  |  |
|  |  | Central | 3.67 (2.76) | 2.06 (1.05) |  |  | 3.05 (1.81) | 1.88 (0.88) |  |  |
|  |  | Parietal | 5.46 (4.38) | 3.03 (1.27) |  |  | 4.34 (2.6) | 2.64 (1.06) |  |  |
| **Theta** | Time 1 | Frontal | 0.91 (0.56) | 0.53 (0.3) | 8.715 | 0.004* | 0.65 (0.3) | 0.41 (0.2) | 17.515 | 0.001* |
|  |  | Central | 0.96 (0.71) | 0.52 (0.35) |  |  | 0.67 (0.34) | 0.4 (0.18) |  |  |
|  |  | Parietal | 1.25 (1.02) | 0.7 (0.45) |  |  | 0.81 (0.42) | 0.53 (0.33) |  |  |
|  | Time 2 | Frontal | 0.87 (0.55) | 0.64 (0.38) |  |  | 0.74 (0.29) | 0.54 (0.26) |  |  |
|  |  | Central | 0.88 (0.75) | 0.54 (0.37) |  |  | 0.72 (0.39) | 0.46 (0.24) |  |  |
|  |  | Parietal | 1.1 (0.97) | 0.75 (0.49) |  |  | 0.93 (0.52) | 0.61 (0.38) |  |  |
| **Alpha** | Time 1 | Frontal | 0.81 (0.57) | 0.57 (0.5) | 3.738 | 0.057† | 0.63 (0.4) | 0.5 (0.62) | 4.589 | 0.035* |
|  |  | Central | 1.07 (0.9) | 0.73 (0.8) |  |  | 0.82 (0.61) | 0.54 (0.42) |  |  |
|  |  | Parietal | 1.59 (1.24) | 1.25 (1.49) |  |  | 1.36 (1.34) | 1 (1.39) |  |  |
|  | Time 2 | Frontal | 0.8 (0.45) | 0.73 (0.67) |  |  | 0.72 (0.46) | 0.58 (0.42) |  |  |
|  |  | Central | 0.99 (0.83) | 0.86 (0.96) |  |  | 0.97 (0.87) | 0.64 (0.59) |  |  |
|  |  | Parietal | 1.41 (1.15) | 1.48 (1.65) |  |  | 1.64 (1.56) | 1.22 (1.48) |  |  |
| **Beta** | Time 1 | Frontal | 0.2 (0.13) | 0.14 (0.06) | 6.514 | 0.012* | 0.16 (0.11) | 0.13 (0.06) | 6.389 | 0.013* |
|  |  | Central | 0.21 (0.13) | 0.13 (0.06) |  |  | 0.17 (0.11) | 0.12 (0.06) |  |  |
|  |  | Parietal | 0.25 (0.14) | 0.17 (0.07) |  |  | 0.21 (0.12) | 0.15 (0.09) |  |  |
|  | Time 2 | Frontal | 0.23 (0.15) | 0.17 (0.07) |  |  | 0.18 (0.1) | 0.13 (0.05) |  |  |
|  |  | Central | 0.22 (0.15) | 0.17 (0.11) |  |  | 0.18 (0.12) | 0.13 (0.06) |  |  |
|  |  | Parietal | 0.26 (0.15) | 0.21 (0.14) |  |  | 0.22 (0.13) | 0.17 (0.08) |  |  |
| **T:B** | Time 1 | Frontal | 5.64 (2.83) | 4.43 (1.99) | 2.456 | 0.121 | 5.34 (2.69) | 4.2 (2.07) | 4.770 | 0.032* |
|  |  | Central | 5.75 (2.88) | 4.45 (1.95) |  |  | 5.26 (2.65) | 4.24 (1.86) |  |  |
|  |  | Parietal | 5.7 (3.03) | 4.36 (1.98) |  |  | 4.95 (2.38) | 4.02 (1.88) |  |  |
|  | Time 2 | Frontal | 4.97 (2.62) | 4.42 (1.57) |  |  | 5.37 (2.51) | 4.66 (1.69) |  |  |
|  |  | Central | 5.54 (3.39) | 4.37 (1.76) |  |  | 5.48 (2.53) | 4.59 (1.76) |  |  |
|  |  | Parietal | 5.23 (3.23) | 4.26 (1.68) |  |  | 5.34 (2.64) | 4.2 (1.82) |  |  |

**p < 0.05; †p < 0.1; T:B theta/beta ratio; µV mean power; SD standard deviation*

# S5. Mean amplitude in µV and standard deviation (SD), prior to transformations, and with age and gender controlled for, in ADHD and control groups across frequency bands and theta/beta ratio at Fz, Cz and Pz

|  |  |  | Delta | Theta | Alpha | Beta | T:B |
| --- | --- | --- | --- | --- | --- | --- | --- |
| Electrode |  |  | µV (SD) | µV (SD) | µV (SD) | µV (SD) | µV (SD) |
|  |  |  |  |  |  |  |  |
| Fz | T1 | Control | 3.23 (2.22) | 0.64 (0.36) | 0.64 (0.53) | 0.18 (0.17) | 0.18 (0.17) |
|  | T1 | ADHD | 4.29 (3.38) | 0.85 (0.62) | 0.8 (0.66) | 0.22 (0.18) | 0.22 (0.18) |
|  | T2 | Control | 4.11 (3.28) | 0.74 (0.4) | 0.71 (0.51) | 0.16 (0.12) | 0.16 (0.12) |
|  | T2 | ADHD | 4.52 (3.62) | 0.85 (0.61) | 0.85 (0.72) | 0.21 (0.18) | 0.21 (0.18) |
|  |  |  |  |  |  |  |  |
|  |  |  |  |  |  |  |  |
| Cz | T1 | Control | 2.07 (1.41) | 0.56 (0.38) | 0.62 (0.47) | 0.19 (0.13) | 0.19 (0.13) |
|  | T1 | ADHD | 3 (3.59) | 0.74 (0.64) | 0.77 (0.75) | 0.19 (0.17) | 0.19 (0.17) |
|  | T2 | Control | 2.59 (1.44) | 0.66 (0.37) | 0.71 (0.68) | 0.19 (0.17) | 0.19 (0.17) |
|  | T2 | ADHD | 2.72 (1.92) | 0.7 (0.55) | 0.78 (0.87) | 0.23 (0.21) | 0.23 (0.21) |
|  |  |  |  |  |  |  |  |
|  |  |  |  |  |  |  |  |
| Pz | T1 | Control | 2.84 (1.85) | 0.7 (0.5) | 1.26 (1.49) | 0.18 (0.13) | 0.18 (0.13) |
|  | T1 | ADHD | 4.3 (3.72) | 0.96 (0.91) | 1.43 (1.49) | 0.2 (0.14) | 0.2 (0.14) |
|  | T2 | Control | 3.58 (3.42) | 0.81 (0.76) | 1.7 (2.39) | 0.18 (0.14) | 0.18 (0.14) |
|  | T2 | ADHD | 4.03 (3.37) | 0.87 (0.85) | 1.38 (1.47) | 0.21 (0.17) | 0.21 (0.17) |
|  |  |  |  |  |  |  |  |

*T:B theta/beta ratio; µV mean power; SD standard deviation; T1 time 1; T2 time 2*

# S6. Significance values and effect sizes for ANCOVA factors and interactions, controlling for age, gender, using data from mid-line electrode (Fz, Cz, Pz).

|  | | Delta | Theta | Alpha | Beta | T:B |
| --- | --- | --- | --- | --- | --- | --- |
| Time | F | 0.00 | 1.60 | 0.90 | 0.01 | 0.14 |
|  | p | 0.984 | 0.207 | 0.345 | 0.905 | 0.905 |
|  | η2 | 0.0001 | 0.0094 | 0.0054 | 0.0001 | 0.0001 |
| Region | F | 2.08 | 4.16 | 4.46 | 0.15 | 0.15 |
|  | p | 0.127 | 0.016* | 0.012* | 0.860 | 0.860 |
|  | η2 | 0.0009 | 0.0248 | 0.0271 | 0.0009 | 0.0009 |
| Group | F | 6.92 | 2.97 | 1.14 | 4.19 | 2.62 |
|  | p | 0.009* | 0.870 | 0.288 | 0.107 | 0.107 |
|  | η2 | 0.0341 | 0.0150 | 0.0066 | 0.0141 | 0.0141 |
| Group*Region | F | 1.63 | 0.82 | 0.97 | 2.14 | 2.14 |
|  | p | 0.198 | 0.440 | 0.380 | 0.120 | 0.120 |
|  | η2 | 0.0133 | 0.0049 | 0.0059 | 0.0133 | 0.0133 |
| Group*Time | F | 3.37 | 4.70 | 1.54 | 0.32 | 0.32 |
|  | p | 0.068† | 0.032* | 0.217 | 0.570 | 0.570 |
|  | η2 | 0.0020 | 0.0274 | 0.0092 | 0.0020 | 0.0021 |

*Activity bands defined as: delta 0.5-3.4Hz, theta 3.5-7.5Hz, alpha 7.5-12Hz, beta 12-30Hz.* ******* *denotes significant at p<0.05. † denotes trend level effect at p<0.08. Effect size (η^2^); 0.0099 constitutes a small effect, 0.0588 a medium effect and 0.1379 a large effect.*

# S7. Significance values and effect sizes for ANCOVA factors and interactions, controlling for age, gender, and IQ, using data from mid-line electrode (Fz, Cz, Pz).

|  | | Delta | Theta | Alpha | Beta | T:B |
| --- | --- | --- | --- | --- | --- | --- |
| Time | F | 0.64 | 0.26 | 0.48 | 0.06 | 0.63 |
|  | p | 0.425 | 0.610 | 0.827 | 0.802 | 0.802 |
|  | η2 | 0.0039 | 0.0015 | 0.0003 | 0.0004 | 0.0004 |
| Region | F | 3.52 | 2.09 | 2.53 | 0.68 | 0.68 |
|  | p | 0.031* | 0.125 | 0.091 | 0.505 | 0.508 |
|  | η2 | 0.0215 | 0.0126 | 0.0156 | 0.0043 | 0.0043 |
| Group | F | 3.74 | 1.57 | 0.98 | 0.70 | 0.70 |
|  | p | 0.055† | 0.212 | 0.324 | 0.403 | 0.403 |
|  | η2 | 0.0191 | 0.0081 | 0.0057 | 0.0039 | 0.0039 |
| Group*Region | F | 0.37 | 0.10 | 0.55 | 0.53 | 0.53 |
|  | p | 0.658 | 0.893 | 0.577 | 0.591 | 0.591 |
|  | η2 | 0.0023 | 0.0006 | 0.0034 | 0.0033 | 0.0033 |
| Group*Time | F | 5.03 | 5.09 | 2.21 | 0.05 | 0.05 |
|  | p | 0.026* | 0.025* | 0.014* | 0.824 | 0.824 |
|  | η2 | 0.0305 | 0.0299 | 0.0132 | 0.0003 | 0.0003 |

*Activity bands defined as: delta 0.5-3.4Hz, theta 3.5-7.5Hz, alpha 7.5-12Hz, beta 12-30Hz.* ******* *denotes significant at p<0.05. † denotes trend level effect at p<0.08. Effect size (η^2^); 0.0099 constitutes a small effect, 0.0588 a medium effect and 0.1379 a large effect.*

# S8. Significance values for ANCOVA factors and interactions, controlling for age and gender, using global field synchronisation scores and showing covariate interaction with the dependent variable.

|  | | Delta | Theta | Alpha | Beta |
| --- | --- | --- | --- | --- | --- |
| Group | F | 1.26 | 0.43 | 0.32 | 0.11 |
|  | p | 0.263 | 0.512 | 0.575 | 0.738 |
| Age | F | 7.93 | 13.80 | 6.83 | 4.63 |
|  | p | 0.005* | 0.000* | 0.010* | 0.033* |
| Gender | F | 6.63 | 6.28 | 0.01 | 0.37 |
|  | p | 0.110 | 0.013* | 0.936 | 0.543 |
| Condition | F | 0.01 | 3.21 | 0.27 | 0.04 |
|  | p | 0.929 | 0.075† | 0.607 | 0.849 |
| Condition*Age | F | 0.00 | 3.44 | 0.41 | 0.27 |
|  | p | 0.964 | 0.066† | 0.523 | 0.870 |
| Condition*Gender | F | 0.20 | 2.37 | 1.05 | 0.27 |
|  | p | 0.653 | 0.126 | 0.308 | 0.606 |
| Condition*Group | F | 1.90 | 2.21 | 1.11 | 1.11 |
|  | p | 0.170 | 0.139 | 0.295 | 0.294 |

*Activity bands defined as: delta 0.5-3.4Hz, theta 3.5-7.5Hz, alpha 7.5-12Hz, beta 12-30Hz.* ******* *denotes significant at p<0.05. † denotes trend level effect at p<0.08.*

# S9. Correlations of age with global field synchronisation.

|  | Delta T1 | Theta T1 | Alpha T1 | Beta T1 | Delta T2 | Theta T2 | Alpha T2 | Beta T2 |
| --- | --- | --- | --- | --- | --- | --- | --- | --- |
| Pearson r | 0.21 | 0.34 | 0.2 | 0.14 | 0.17 | 0.15 | 0.15 | 0.12 |
| p | 0.006* | <0.0001* | 0.009* | 0.068† | 0.029* | 0.062† | 0.052* | 0.132 |

*Activity bands defined as: delta 0.5-3.4Hz, theta 3.5-7.5Hz, alpha 7.5-12Hz, beta 12-30Hz.* ******* *denotes significant at p<0.05. † denotes trend level effect at p<0.08.*
